# Bicelle-induced skin penetration mechanism for hydrophilic molecules

**DOI:** 10.1039/d5ra05449d

**Published:** 2025-08-11

**Authors:** Yusuke Hayashida, Kazuhiro Ohata, Liliana de Campo, Mina Tanigawa, Noriko Miyamoto, Takuya Matsunaga, Mina Sakuragi

**Affiliations:** a Faculty of Engineering, Department of Nanoscience, Sojo University 4-22-1 Ikeda, Nishi-ku Kumamoto City 860-0082 Japan d08b0101@nano.sojo-u.ac.jp; b Australian Centre for Neutron Scattering (ACNS), Australian Nuclear Science and Technology Organization (ANSTO) Sydney NSW 2234 Australia; c Department of Applied Chemistry, Faculty of Engineering, Aichi Institute of Technology 1247, Yachigusa, Yakusa-cho Toyota Aichi 470-0392 Japan; d Department of Chemistry and Biochemistry, University of Kitakyushu 1-1 Hibikino, Wakamatsu-ku Kitakyushu Fukuoka 808-0135 Japan

## Abstract

In this study, we demonstrated that the disk-shaped structures, bicelles, composed of 1,2-dipalmitoyl-*glycero*-3-phosphocholine (DPPC) and 1,2-diheptanoyl-*sn*-glycero-3-phosphocholine (DHPC) enhance the transdermal delivery of hydrophilic and high-molecular-weight compounds by forming water-containing lamellar structures within the skin barrier, stratum corneum (SC). Skin permeation studies using fluorescent probes and cy3 modified RNA oligonucleotide revealed that bicelle pretreatment significantly enhanced their skin penetration. Furthermore, the interaction mechanism between bicelles and SC was elucidated using small-angle X-ray scattering (SAXS) and small-angle neutron scattering (SANS). The results showed that bicelles collapsed within the SC and reorganized into hydrated lamellae primarily composed of DPPC, which served as the new permeation pathway. Unlike DPPC vesicles, bicelles enhanced skin permeability without disrupting the original lamellar structures of intercellular lipids in the SC, thereby maintaining the skin barrier function. These findings reveal a novel mechanism of bicelle-mediated skin penetration and highlight their potential as safe and effective carriers for transdermal drug delivery.

## Abstract 2

We demonstrated that the disk-shaped structures, bicelles, composed of phospholipids enhance the transdermal delivery of hydrophilic and high-molecular-weight compounds by forming water-containing lamellar structures within the stratum corneum (SC).

## Graphical abstract

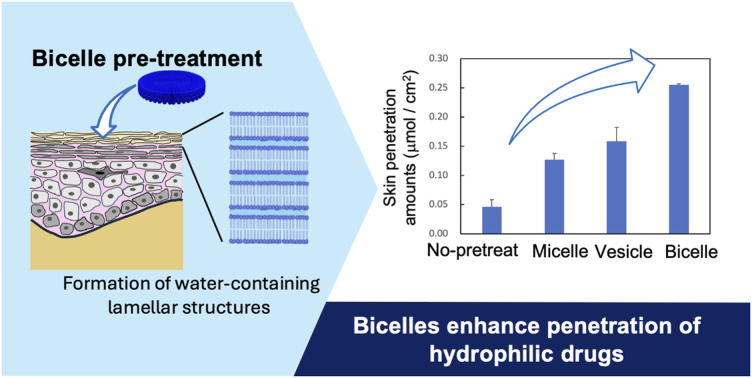

## Introduction

Hydrophilic and high-molecular-weight drugs, such as biopharmaceuticals, cannot permeate the skin barrier, the stratum corneum (SC). The SC comprises corneocytes and intercellular lipids, and hydrophobic substances with low molecular weight cross the intercellular lipids. To improve skin permeability of biopharmaceuticals, chemical approaches using drug carriers and skin penetration enhancers,^1,2^ as well as physical methods such as microneedles and iontophoresis employing electrical energy, have been investigated.^3,4^ However, many of these conventional techniques enhance permeability by disrupting the SC structure. Recently, ionic liquids and deep eutectic solvents composed of biocompatible components have attracted attention as novel skin penetration enhancers for biopharmaceuticals.^2,5–7^ However, they may disturb the structural integrity of SC or extract lipids.^7,8^ Therefore, long-term use in patients with skin disorders is limited.

To address these limitations, we focused on the bicelles, disk-shaped structures composed of phospholipids. Bicelles are disrupted in the SC and cannot penetrate beyond the SC into the deep skin layers.^9^ Accordingly, bicelles are useful for retaining substances inside the intercellular spaces of SCs.^10,11^ However, few studies report on the use of bicelles to deliver drugs deep into the skin. We previously reported that bicelles dispersed in deep eutectic solvents^12^ or arginine peptide-containing bicelles^13^ can enhance the transdermal delivery of encapsulated drugs compared with conventional bicelles. Lubio *et al.* reported that the skin permeability of anti-inflammatory drugs is enhanced after bicelle treatment;^14^ however, the underlying mechanism of action remains unclear. In addition, to our knowledge, the application of bicelles for the transdermal delivery of hydrophilic macromolecules, such as biopharmaceuticals, has not been explored. In this study, we compared the skin permeation of hydrophilic and hydrophobic compounds following bicelle application and investigated how bicelles interact with SC lipids using small-angle X-ray scattering (SAXS) and small-angle neutron scattering (SANS). Bicelles also enhanced RNA oligonucleotide skin permeation. Our findings demonstrate that bicelles reorganize into hydrated lamellar structures within the SC, potentially serving as permeation pathways for hydrophilic drugs.

## Experimental

### Material

1,2-Dipalmitoyl-*sn*-glycero-3-phosphocholine (DPPC) was purchased from NOF. 1,2-Diheptanoyl-*sn*-glycero-3-phosphocholine (DHPC) was purchased from Avanti Polar Lipid. Fluorescein sodium was purchased from Kanto Chemical. Fluorescein was purchased from Tokyo Chemical Industry. Isopropyl myristate (IPM) was obtained from Tokyo Chemical Industry. Trypsin and trypsin inhibitor were purchased from FUJIFILM Wako Pure Chemicals. Full-thickness skin of 7 week-old male hairless mice (Hos: HR-1, thickness: approximately 0.5–0.7 mm) with the subcutaneous fat removed was purchased from Japan SLC. Cy3-labeled siRNA (5′-Cy3-GGCCUUUCACUACUCCUACTT-3′) was provided by Genedesign (Osaka, Japan). Hairless male mice (Hos: HR-1, 25–35 g; 7 weeks old) were obtained from SLC Co., Ltd (Shizuoka, Japan). They were housed in a temperature-controlled standard animal room under a 12 h light-dark cycle. Animal housing and experimental procedures were conducted in accordance with the guidelines of the National Institutes of Health and were approved by the Animal Experimentation Committee of Sojo University (approval numbers: 2025-P-025).

### Bicelle, DPPC dispersion, and DHPC solution preparation

Bicelles were prepared using DPPC and DHPC as the long-and short-chain phospholipids, respectively.^15^ DPPC and DHPC were weighed and dissolved in chloroform. The lipid solutions were mixed in a 10 mL eggplant flask at a DPPC : DHPC molar ratio of 2 : 1. The mixed solution was evaporated using a rotary evaporator to form a thin lipid film, which was then vacuum-dried. Water was then added to the lipid film and cycles of bath-type ultrasonication and freezing with liquid nitrogen were repeated until a clear solution was obtained. The final lipid concentration was adjusted to 20 wt%. The pH of the resulting bicelle solution was adjusted to 7.0 using NaOH and HCl solutions. The DPPC dispersions and DHPC solutions were prepared using the same procedure as for bicelle preparation, with concentrations adjusted to match the respective concentrations of DPPC or DHPC in the bicelle solution.

### Trans-epidermis water loss (TEWL) measurements *in vitro*

The full-thickness hairless mouse skin was cut into small pieces measuring approximately 3 × 3 cm^2^. Each sample was added to a skin sample in a Petri dish. After 24 h, the skin was washed thrice with water, and excess sample was removed by absorption with a laboratory wipe. The TEWL of each skin sample was measured using a Tewameter (TM Hex; Courage; Khazaka Electronic GmbH, Cologne, Germany).

### TEWL measurements *in vivo*

For the *in vivo* experiment, TEWL was measured on the back of mice under anesthesia using a Tewameter to record the values before bicelle pretreatment. Next, 100 μL of bicelle was applied to the back of the mice, and a 1 cm square gauze pad was placed on top and secured with a patch. After 24 h, the mice were anesthetized, and the patch and gauze pad were removed. TEWL was then measured at the site where the bicelle was applied.

### Dynamic light scattering (DLS) measurements

A DelsaMax Light Scattering Analyzer (Beckman Coulter) operating at a wavelength of 532 nm and a scattering angle of 90° was used for DLS. A 45 μL transparent cell with a path length of 1 cm was used for the measurements. All samples were analyzed at a constant temperature of 25 °C. The resulting data are presented as histograms, and the average sizes of the formed bicelles, vesicles, micelles, and aggregates are determined and plotted.

### Skin penetration experiments

Skin penetration experiments were conducted using Franz diffusion cells (PermeGear, Inc., USA) with a diameter of 5.0 mm. A solvent mixture of pH 7.4 phosphate buffer, propylene glycol, and ethanol in a 6 : 2 : 2 ratio was placed in the receptor chamber, which was continuously stirred at 600 rpm and maintained at 37 °C. Then, 50 μL of a bicelle, DPPC, or DHPC solution was applied to the SC surface. After 24 h, the sample solution on the skin was removed and the skin was wiped with a Kimwipe moistened with 70% aqueous ethanol solution. Next, 1 mg mL^−1^ fluorescein sodium aqueous solution, fluorescein dissolved in IPM, or 100 μM of cy3-modified RNA dissolved in PBS was applied to the SC surface. The concentration of the fluorescent substances in the receptor chamber was analyzed using a fluorescence spectrometer (RF-6000, Shimadzu). In the RNA skin penetration test, considering the possibility that degraded RNA could pass through and increase the fluorescence intensity, the receptor solution was purified in an ultrafiltration unit (Vivaspin® 2 Centrifugal Concentrator Hydrosart®, 2k MWCO). No fluorescence was observed in the filtrate, indicating that RNA was not degraded. The skin penetration was reported as the mean ± standard deviation (SD) (*n* = 3). Significant differences among the samples were determined using a one-tailed unpaired Student's *t*-test. Following the skin penetration test, skin samples were immersed in formalin for several hours. The samples were then cryosectioned using a cryostat (Leica CM1950), and the resulting cross sections were examined using a fluorescence microscope (KEYENCE BZ-X810).

### Small-angle X-ray scattering (SAXS)

Beamline 40B2 of Spring-8, a synchrotron radiation facility in Japan, was used to conduce SAXS. X-ray diffraction profiles were obtained using a Pilatus detector for SAXS. The X-ray wavelength was 0.1 nm, and the sample-to-detector distance (SDD) was set at approximately 2000 mm. SAXS was used to examine the bicelles, DPPC dispersion, and DHPC solution structures. Exposure time was 60 s. The samples were measured using a quartz cell capillary with a diameter of 2 mm.

The skin samples were immersed overnight at 4 °C in a 0.1% trypsin solution prepared in a 10 mM phosphate buffer (pH 7.4). The SC was then separated from the skin by incubation at 37 °C for 4 h. Following separation, the SC was immersed in a 0.1% trypsin inhibitor solution and rinsed with water. Although trypsin may slightly affect some skin components, the intercellular lipid lamellar structures of the SC are highly stable against such enzymatic treatment given their tightly packed and ordered nature.^16^ Subsequently, five layers of SC were stacked onto the PEEK film and dried overnight in a vacuum pump. Dry SC samples were placed on a PEEK film, which was then placed in a custom sample cell designed by the Special Research Laboratory at the University of Kitakyushu, Faculty of Environmental Engineering (Machine Center). The scattering profiles of the SC were recorded before sample application, 1 min after application, and several times thereafter for up to 24 h.

We prioritized the use of identical sample conditions for both SAXS and SANS measurements. Therefore, we employed dry SC samples in both cases, even though hydration-dependent changes in the structural characteristics of the SC lamellae have been previously reported.^17^ For SANS, we aimed to maximize contrast from deuterated carriers and therefore avoided the addition of H_2_O or D_2_O to the SC samples.

### Small-angle neutron scattering (SANS)

SANS measurements were performed to observe the structural changes in bicelles, DPPC dispersion, and DHPC solution within the SC using the BILBY instrument^18^ at the Australian Centre for Neutron Scattering, Australian Nuclear Science and Technology Organization (ANSTO) Lucas Heights, NSW, Australia.

For each kinetic sample, nine stacked dry SC samples on the PEEK film were transferred to cells with quartz windows and a path length of 1 mm and held with a Teflon spacer with a opening of 10 mm diameter. First, the scattering pattern of the dry SC was obtained. Each lipid assembly sample (100 μL) was then applied to the SC, and the scattering patterns were recorded and subsequently sliced at 20 min time intervals. Subsequent measurements were performed after 1 day. The exposure time for all other samples was 20 min, and all measurements were performed at 25 °C.

The SDD was set to 8 m for the rear detector and 1.8 and 2.8 m for the curtain detectors, and the neutron wavelength was set to 0.6 nm with a wavelength distribution (Δ*λ*/*λ*) of 10% full width at half-maximum. The solutions were measured with a 12.5 mm aperture and the SC samples with an 8 mm Cd aperture were placed directly on the sample cells. Data were reduced using custom Python scripts in the Mantid suite,^19^ placed on an absolute scale using an empty beam transmission measurement, and background-subtracted using the solvent for the micellar solutions and the empty cell for the kinetic samples.

### SAXS and SANS profile fitting analysis

The obtained SAXS and SANS profiles of the bicelles were fitted to the following theoretical equation for a core–shell disk^20^ using SAS View 5.0:
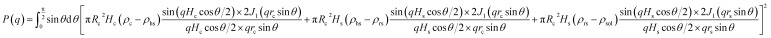


The SAXS and SANS profiles of the DHPC solution were fitted to the following theoretical equation for a core–shell sphere^21^ using SAS View 5.0:
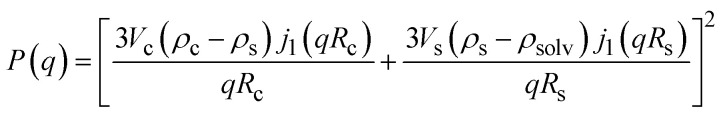
where 
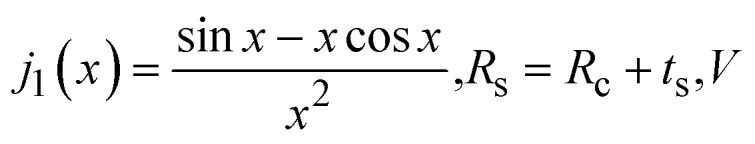
 and *ρ* indicate volume and SLD, respectively. Subscripts c and s denote the core and shell regions of the micelles, respectively.

## Results and discussion

The structural characteristics of the prepared samples were evaluated using SAXS and SANS. The SAXS profiles of the bicelles and DHPC solutions showed convex curves, characteristic of a form factor, whereas the profile of the DPPC dispersion showed three peaks in the form factor scattering curve (Fig. S1a). The appearance of the peak at the 1 : 2 : 3 ratio indicates the presence of a lamellar structure. From the peak position, the interlamellar spacing was calculated to be *d* = 6.37 nm, suggesting that DPPC formed multilamellar structures, likely multilamellar vesicles. The experimental scattering profiles of the bicelles and DHPC solution were well fitted by theoretical models of core–shell disks and core–shell spheres, respectively, indicating that bicelles adopt a disk-shaped structure, as previously reported,^13,15^ whereas DHPC forms spherical micelles. Furthermore, similar structural features were confirmed when the solvent was changed to D_2_O, as shown by fitting of the SANS data, which yielded profiles consistent with those from SAXS (Fig. S1b). The best-fit parameters for the fitting analysis are shown in Tables S1 and S2. For DPPC dispersed in D_2_O, the periodicity of lamellar is 6.95 nm, which is slightly larger than the SAXS results (*d* = 6.37 nm), because the extent of lipid interactions shows slight differences between aqueous and deuterated environments. Dynamic light scattering (DLS) showed a single peak at 11.1 nm for bicelles, whereas DPPC vesicles exhibited two peaks at 86 nm and 532 nm, and DHPC micelles exhibited two peaks at 4.4 nm and 396 nm (Fig. S2). Although the DLS data for DHPC show strong scattering signals from large particles, the scattering intensity scales with the sixth power of the particle size, suggesting that such aggregates are present only in small quantities.

**Fig. 1:**
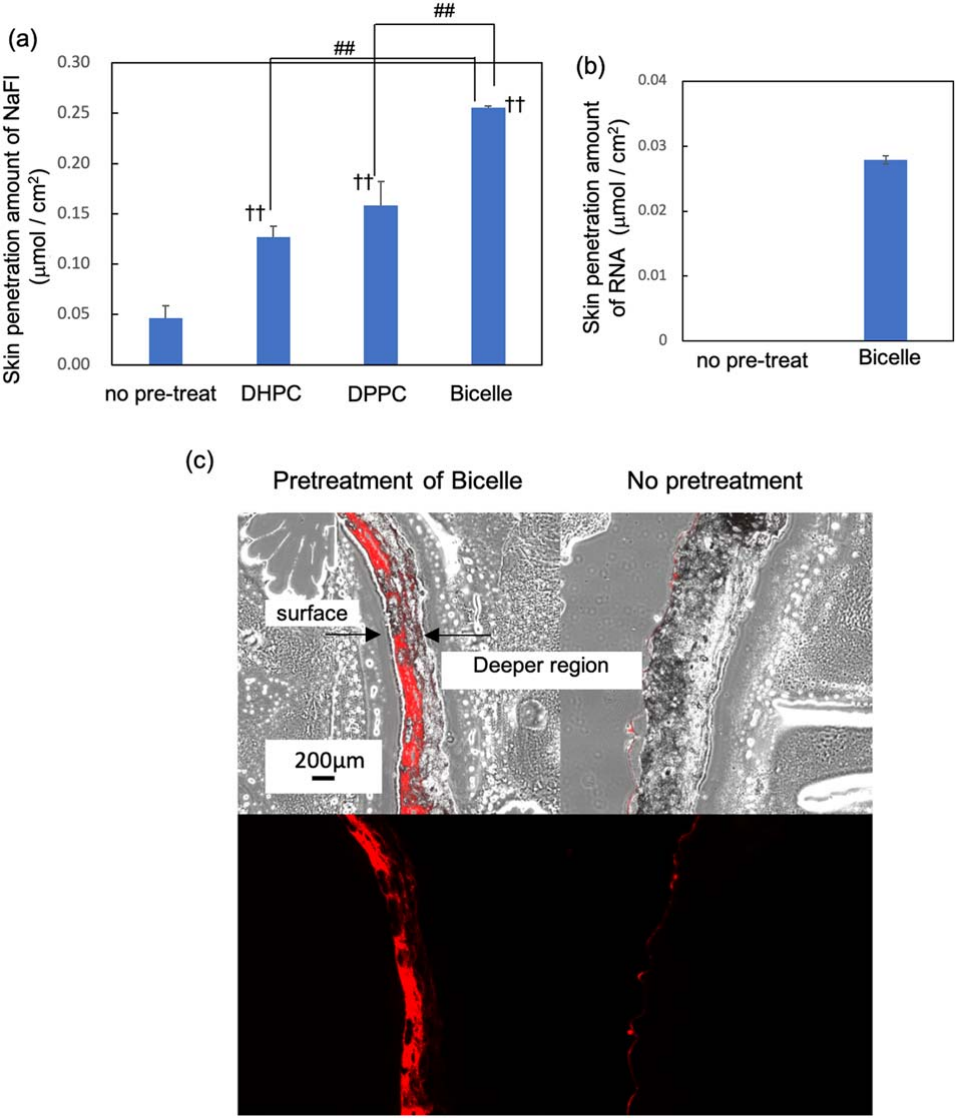
Averaged skin penetration amounts of (a) NaFl and (b) RNA. Data are the means ± standard deviations (SDs) of three experiments. ††*p* < 0.01 *vs.* control; ##*p* < 0.01 *vs.* bicelle treatment. (c) Fluorescence microscopy images and merged images with bright-field microscopy showing the localization of cy3 in skin.

Subsequently, *in vitro* skin penetration tests of low-molecular-weight compounds were conducted using various formulations, including skin penetration enhancers, bicelles, DPPC vesicles, and DHPC micelles. The skin of hairless mice was used. Fluorescein (Fl) dissolved in the oil phase, isopropyl myristate (IPM) can permeate the skin without pre-treatment with lipid-based structures because of its hydrophobicity and low molecular weight. When the skin was pre-treated with bicelles, DPPC vesicles, or DHPC micelles prior to the application of Fl in the IPM, permeation remained nearly unchanged compared to that without pretreatment (Fig. S3). However, when a hydrophilic sodium fluorescein (NaFl) solution, was applied to the skin after lipid structure pre-treatment, its skin penetration was significantly enhanced compared to that without pre-treatment (Fig. 1a). NaFl skin penetration enhancement was the highest with bicelle pretreatment, followed by DPPC and DHPC. We also evaluated the Cy3-labeled double-stranded RNA (molecular weight: 13.4 kDa). RNA did not penetrate the skin without pre-treatment, but pre-treatment with bicelles enabled the RNA to permeate through 500–700 μm-thick hairless mouse skin (Fig. 1b). Fluorescence microscopy images of the skin sections clearly showed strong Cy3-derived fluorescence following bicelle pre-treatment beyond the epidermis and into the dermis (Fig. 1c). The following reasons may explain why the Cy3-RNA fluorescence signal did not span the full tissue thickness (Fig. 1c), even though it was observed in the receptor cell (Fig. 1b). First, skin permeation may occur *via* limited intercellular routes, resulting in localized, rather than uniform, fluorescence signals. Second, the outer skin layers are highly hydrophobic and are considered to be a rate-limiting barrier. Thus, RNA may accumulate there, whereas in the deeper dermal regions (which are more aqueous), permeation may proceed more readily. By 24 h, the RNA in the deep dermis may have already diffused into the receptor compartment and no longer be retained in the deep skin layer, leading to weaker fluorescence in those layers. These findings indicate that bicelle pre-treatment dramatically enhances the skin permeability of hydrophilic substances and enables transdermal macromolecule delivery. These findings represent a groundbreaking approach with potential applications for delivering hydrophilic biopharmaceuticals.

**Fig. 2:**
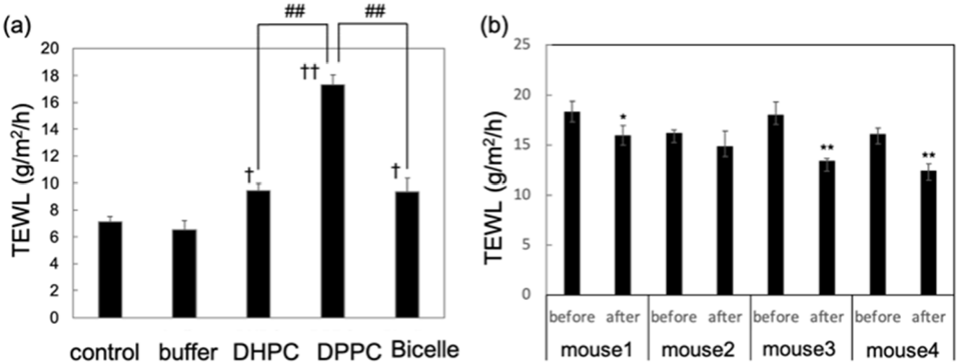
Trans-epidermal water loss (TEWL) values at 24 h after applying each solvent to the skin (*n* = 3) *in vitro* (a) and *in vivo* (b). ‘Control’ represents the TEWL values of the skin without any treatment. ‘Buffer’ represents the values of the skin after applying phosphate buffer. †*p* < 0.05, ††*p* < 0.01 *vs.* control. ##*p* < 0.01 *vs.* 1,2-dipalmitoyl-*glycero*-3-phosphocholine (DPPC).

Fig. 2 shows the trans-epidermis water loss (TEWL) measurements values. TEWL is an indicator of water evaporation through the epidermis, and increases when the skin barrier is damaged. Bicelle treatment caused a slight increase in TEWL compared to that in the control, and DHPC showed a similar trend (Fig. 2a). In contrast, DPPC vesicles, which have lower skin penetration-enhancing effects than bicelles, clearly increased TEWL. However, this value does not reach the moderate damage level of 30 g m^2^ h^−1^,^22^ even DPPC vesicles can be considered to cause only mild damage. Notably, although skin barrier disruption tends to enhance transdermal penetration, in our study, bicelles promoted significantly higher skin permeation than DPPC vesicles without causing skin barrier damage. These results indicate that bicelles and DPPC vesicles may enhance skin permeability *via* different mechanisms, as reflected by their different effects on TEWL. Furthermore, *in vivo* experiments, TEWL values decreased to approximately 82.6% ± 8.2% of the pre-treatment levels after bicelle application, suggesting that bicelles improve the skin barrier (Fig. 2b). Indeed, as pointed out by Atwood *et al.*,^23^*in vitro* and ex vivo skin models lack system-level components, such as vasculature, immune responses, and innervation, which are present in native skin. Consequently, they cannot fully replicate the dynamic biological responses observed *in vivo*. Furthermore, Grubauer *et al.*^24^ demonstrated that an increase in TEWL triggers epidermal lipid synthesis and barrier repair *in vivo*, a process that does not occur efficiently in isolated skin. Therefore, the discrepancies observed between our *in vitro* and *in vivo* TEWL data for bicelles likely reflect these fundamental physiological differences. We further investigated this mechanism at the molecular level using SAXS and SANS.

SC are composed of corneocytes and intercellular lipids. Generally, substances such as drug carriers and drugs penetrate intercellular lipid regions. These intercellular lipids contain short lamellar structures with repeat distances of approximately 6 nm, long lamellar structures with repeat distance of 13.4 nm, and disordered domains.^17,25^ The short lamellar phase retained water molecules in the hydrophilic regions of the lipids, whereas the long lamellar phase did not.^17^ SAXS detects only the diffraction peaks derived from the long lamellar structures in both dry SCs and SCs containing excess water.^17^

**Fig. 3:**
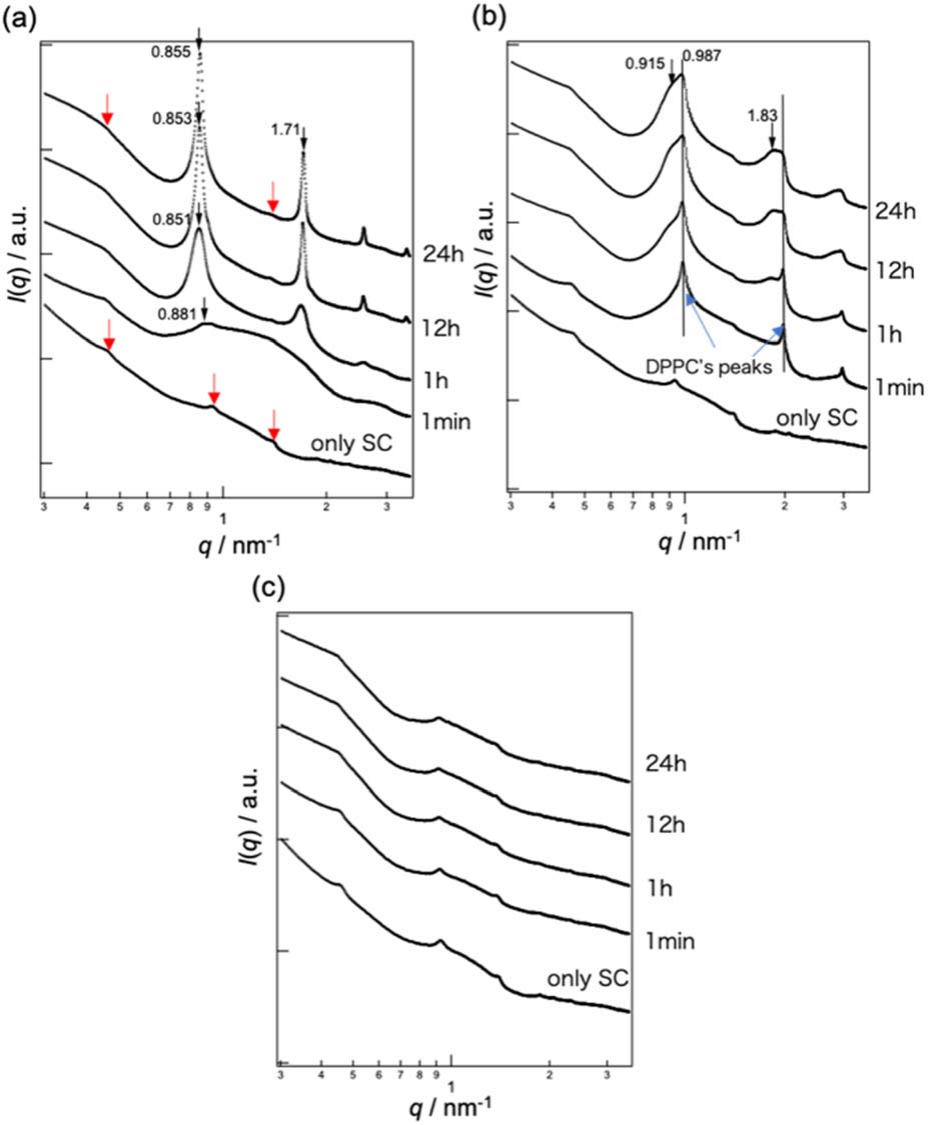
SAXS profiles of the stratum corneum (SC) when applying (a) bicelles, (b) 1,2-dipalmitoyl-*glycero*-3-phosphocholine (DPPC) vesicles, and (c) 1,2-diheptanoyl-*sn*-glycero-3-phosphocholine (DHPC) micelles over time. Red arrows indicate the peaks derived from the SC lamellar structure.

The bottom curve in Fig. 3a shows the SAXS profile of dry SC, in which three peaks attributed to the long lamellar structure were observed, as indicated by the red arrows. A repetition distance of 13.6 nm was calculated from the peak positions. The second curve from the bottom in Fig. 3a shows the SAXS profile of the SC after bicelle application, with each successive curve representing a time point. For SAXS profile of SC at 1 min after applying bicelles, a new peak appeared at *q* = 0.881 nm^−1^ on a convex curve derived from a form factor of bicelle. As time progressed, the form factor of the bicelles gradually disappeared, while the peak at *q* = 0.881 nm^−1^ shifted toward lower *q* values, increased in intensity, and higher-order peaks appeared, indicating the formation of an ordered lamellar structure. This indicates that bicelles collapse within the SC, leading to lamellar structure formation whose regularity becomes increasingly ordered over time. Twenty-four hours after bicelle application, a lamellar structure with a peak at *q* = 0.855 nm^−1^ (*d* = 7.35 nm) was observed. The peak originating from the long lamellar structure of the SC was still detectable even after 24 h.

Barbosa-Barros *et al.* reported that bicelles transform into vesicles or disintegrate to form lamellar structures in the intercellular lipid space of the SC, as observed by cryo-scanning electron microscopy (SEM).^26^ However, they also noted that the SC samples were washed before electron microscopy; thus, the observed structural changes may have been caused by the sample preparation process. In this study, bicelles were directly observed in real-time using SAXS, and the appearance of peaks corresponding to lamellar structures was confirmed. This indicated that the lamellae observed in their cryo-SEM study were not artifacts resulting from dilution during washing, but rather that the bicelles became destabilized in the SC, leading to structural disruption and vesicle formation or newly formed lamellae. Furthermore, real-time SAXS measurements revealed that lamellar structures began forming within 1 min of bicelle application and their structural regularity increased over time.

Fig. 3b shows the time-dependent SAXS profiles of the SC after DPPC vesicle application. The two peaks marked by vertical lines in Fig. 3b correspond to the multilamellar structures of DPPC, with a repeat distance of 6.36 nm. A new peak derived from a lamellar structure appeared on the lower-*q* region of the DPPC-derived peak at *q* = 0.987 nm^−1^ 1 h after application. The repeat distance of this newly formed lamellar structure was 6.86 nm based on the peak position. The DPPC-derived, newly formed lamellar, and SC lamellar peaks were observed after 24 h. In contrast, no structural changes in the SC lamellae were detected upon DHPC micelles application (Fig. 3c).

**Fig. 4:**
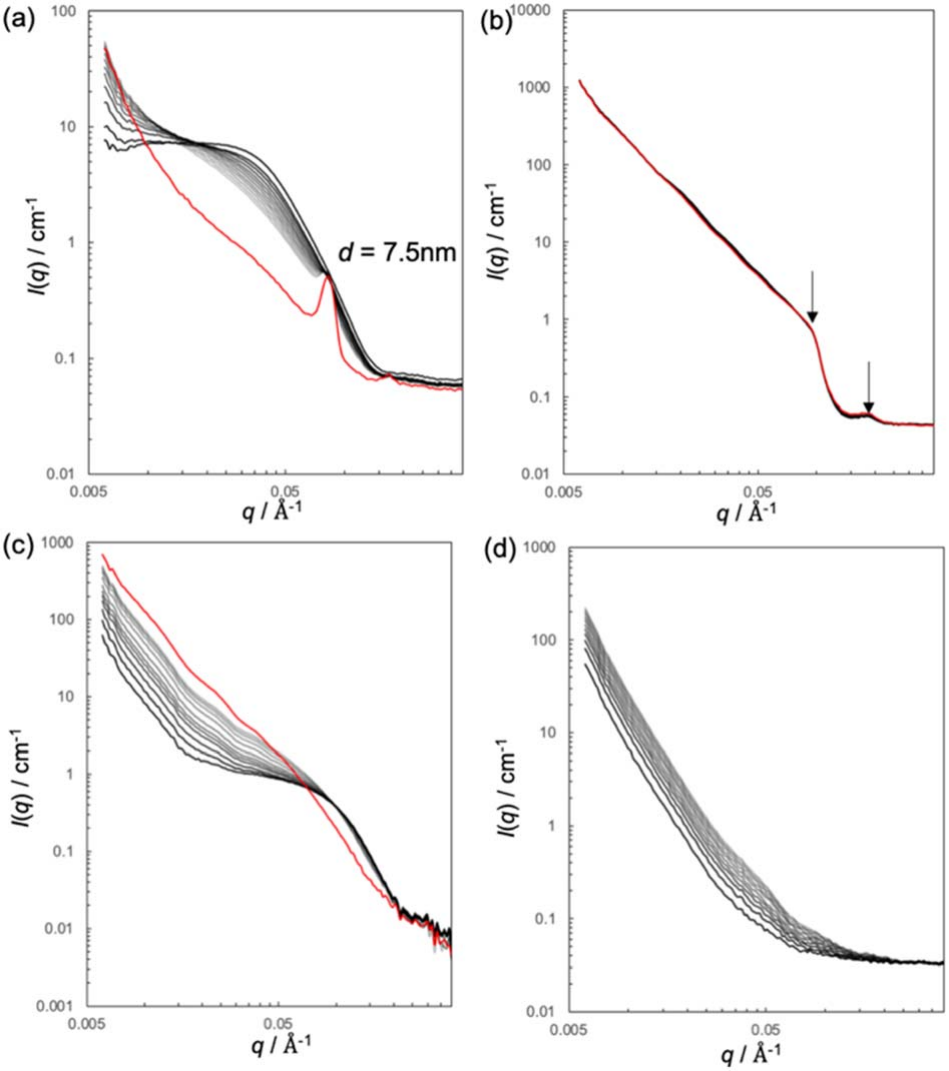
SANS profiles of the stratum corneum (SC) before and after (a) bicelles, (b) 1,2-dipalmitoyl-*glycero*-3-phosphocholine (DPPC) vesicles, (c) 1,2-diheptanoyl-*sn*-glycero-3-phosphocholine (DHPC) micelles, and (d) D_2_O application. Sample solvents were D_2_O. Profiles from immediately after application up to 4 h are shown in black to grey, while the profile at 24 h post-application is shown in red.

SAXS analysis revealed that among the three lipid assemblies (bicelles, DPPC vesicles, and DHPC micelles), bicelles and DPPC vesicles interacted with SC lipids, resulting in structural changes in either the SC lipids or the applied lipid assemblies. However, whether these changes originate from the SC lipids, applied structures, or both remains unclear. To address this uncertainty, SANS measurements were performed. Fig. 4 shows the SANS profiles of the SC after application of (a) bicelles, (b) DPPC vesicles, and (c) DHPC micelles dispersed in D_2_O. The red curves represent the profiles obtained one day after lipid application in each figure. No distinct peaks were observed for the SC alone (Fig. 4d). Therefore, in the SANS measurements, the scattering from SC lipids can be considered negligible, allowing observation of only the scattering derived from the applied lipid assemblies and D_2_O behaviour within the SC.

When bicelles were applied to the SC (Fig. 4a), the form factor originating from the bicelles gradually disappeared over time, and new lamellar structures were formed, which was also previously observed in the SAXS results. After 24 h, a distinct lamellar peak with a repeated distance of 7.5 nm was detected. This repeat distance was similar to that observed using SAXS (*d* = 7.35 nm). In this case, the scattering contrast is proportional to the scattering length density (SLD) between the lipid and solvent. As shown in Table S1, the SLD of the head group of lipids was approximately 1.8 × 10^−6^ Å^−2^ and, that of hydrocarbon chains was −0.28 × 10^−6^ Å^−2^, and that of D_2_O was 6.28 × 10^−6^ Å^−2^. Thus, because the SLD of D_2_O is notably higher than that of the lipids, the lamellar peak is considered to arise from the penetration of the hydrophilic region of the newly formed lamellae. These results suggest that bicelles permeated into the SC, collapsed, and the D_2_O used as the solvent for the bicelles became incorporated into the interlamellar spaces of the newly formed lamellae.

In addition, a similar investigation was conducted using bicelles prepared with deuterated lipids (d-DPPC or d-DHPC), in which the hydrophobic tails were deuterated and the bicelles were dispersed in H_2_O. As shown in Fig. S4a, when d-DPPC/d-DHPC dispersed in H_2_O was applied, a lamellar peak similar to that observed with bicelles dispersed in D_2_O also appeared. A similar result was also observed when using bicelles composed of only deuterated DPPC and non-deuterated DHPC (d-DPPC/h-DHPC) dispersed in H_2_O (Fig. S4b). In contrast, when using bicelles composed of non-deuterated DPPC and deuterated DHPC (h-DPPC/d-DHPC) dispersed in H_2_O, although bicelle disintegration was observed, no peaks corresponding to lamellar structures were detected (Fig. S4c). These findings indicated that the bicelles interacted with intercellular lipids in the SC, leading to their collapse, and that bicelle-derived DPPC formed new lamellar structures, which incorporated water into the hydrophilic regions between the lamellae.

When DPPC dispersed in D_2_O was applied to the SC, peaks corresponding to DPPC multilamellar structures with a repeat distance of 6.95 nm were observed (Fig. 4b). The SANS profiles remained unchanged over the course of one day. The SC of d-DPPC dispersed in H_2_O was measured using SANS (Fig. S5). Almost no changes were observed. These results indicate that the DPPC vesicles neither permeated into the SC nor underwent any structural changes. In the SAXS measurements, although the lamellar structure of DPPC itself remained after its application, the appearance of a new peak at a different position, especially when considered alongside the SANS results, suggested that this new peak originated from intercellular lipids in the SC. This observation suggests that the contact between the DPPC vesicles and the surface lipids of the SC induced a rearrangement of the SC lipids, leading to new lamellar structure formation.

The SANS profile of the SC after DHPC application dispersed in D_2_O showed that while the micellar structure collapsed, no new peaks were observed (Fig. 4c). Combined with the SAXS results, these results indicate that DHPC disintegrates within the SC but does not induce any structural changes in the SC lipids. For comparison, when only D_2_O was applied to the SC, no incorporation of D_2_O into the lamellar structures was observed; instead, only an increase in scattering intensity in the low-*q* region was detected (Fig. 4d). This phenomenon was consistent with the SANS results reported by Charalambopoulou *et al.*^27^ when the SC was exposed to a D_2_O/H_2_O vapor atmosphere. According to their analysis, water was absorbed by corneocytes in the SC upon exposure to excess water vapor. The increase in the scattering intensity in the low-*q* region observed in this study was likely due to the enhanced scattering contrast of corneocytes caused by water uptake. In our previous study, we observed the same phenomenon using SAXS when water was applied to the SC.^7^ These results suggest that incorporating water into the lamellae formed within the SC was observed only in the case of bicelle applications and that this phenomenon may contribute to the enhanced penetration of hydrophilic substances through the SC. This behaviour may be attributed to the high bicelle concentration (20 wt%) and the fact that dry SC absorb water upon exposure. As a result of water uptake by the SC, the water content in the formulation likely decreased, causing bicelle aggregation and a phase transition into lamellar structures composed of DPPC. Therefore, we hypothesized that the structural changes observed in the SC region may reflect transformations of the bicelles themselves rather than alterations in the native SC lipid structures.

Although DPPC application also induced structural rearrangement of the intercellular lipids in the SC, which improved the skin permeability of substances, it likely caused an increase in TEWL due to SC lamellar structure disruption, indicating deterioration of skin barrier function. In contrast, after bicelle application, the newly formed lamellae were primarily composed of DPPC from the bicelles, and the native SC lamellar structures, which play a crucial role in the skin barrier, remained unchanged. Therefore, the skin barrier function was largely maintained.

## Conclusions

In this study, we demonstrated that phosphatidylcholine-based bicelles significantly enhance the transdermal delivery of hydrophilic and high-molecular-weight compounds by inducing the formation of novel water-containing lamellar structures within the SC. Permeation studies revealed that pretreatment with bicelles significantly increased the skin permeation of the low-molecular-weight compound sodium fluorescein. Furthermore, although RNA oligonucleotides did not permeate the skin at all without bicelle pretreatment, their skin permeation became detectable after bicelle application. These results suggest that bicelles play a critical role in enhancing the overall skin delivery of both small and macromolecular hydrophilic compounds. SAXS and SANS measurements further elucidated the mechanism by which bicelles interact with SC lipids. Upon application, the bicelles reorganized into hydrated lamellae primarily composed of DPPC within the SC, forming alternative permeation pathways without disrupting the original lamellar structure. This is in sharp contrast to traditional penetration enhancers, which often damage the skin barrier. Consistent with this, TEWL measurements indicated that bicelle application did not impair the skin barrier function.

Bicelles can serve as promising, biocompatible enhancers for the transdermal delivery of sensitive hydrophilic drugs, including biopharmaceuticals. This study lays the foundation for developing next-generation skin delivery platforms that combine safety with efficacy, and highlights the importance of structural analysis in understanding skin permeation mechanisms.

## Author contributions

YH: data curation, experiments, structural analysis, investigation, methodology, writing – original draft. KO: data curation and experiments. LD: experiments, methodology, review, and editing. MT: experiments and investigation NM: experiments and methodology. TM: experiments and methodology. MS: investigation, methodology, funding acquisition, project administration, resources, supervision, writing – original draft, review, and editing.

## Conflicts of interest

The authors declare a competing financial interest, and a Japanese patent application (no. 2025-7759) has been filed covering part of the data presented (Fig. 1b, c, and 2b).

## Supplementary Material

RA-015-D5RA05449D-s001

## Data Availability

All data supporting this article are included in the main text and the SI. Supplementary information contains SAXS/SANS analysis of the sample solutions, DLS results, skin penetration results, and SANS measurement for bicelles and DPPC applied to the stratum corneum. See DOI: https://doi.org/10.1039/d5ra05449d.
