# Supplementary material for: Bicelle-induced skin penetration mechanism for hydrophilic molecules
Source: RSC Adv. 2025 Aug 11;15(35):28367–74. doi: 10.1039/d5ra05449d (PMC12376770; doi:10.1039/d5ra05449d)
Supplement: RA-015-D5RA05449D-s001 [file RA-015-D5RA05449D-s001.pdf]

Supplementary Information (SI) for RSC Advances.
This journal is © The Royal Society of Chemistry 2025

Supporting Information

Bicelle-Induced Skin Penetration Mechanism for Hydrophilic Molecules

Yusuke Hayashida[a], Kazuhiro Ohata[a], Liliana de Campo[b], Mina Tanigawa[a], Noriko Miyamoto[c], Takuya Matsunaga[d], and Mina Sakuragi *[a]

[a]Faculty of Engineering, Department of Nanoscience, Sojo University, 4-22-1 Ikeda, Nishi-ku, Kumamoto City 860-0082, Japan

[b]Australian Centre for Neutron Scattering (ACNS), Australian Nuclear Science and Technology Organization (ANSTO), Sydney, NSW 2234, Australia

[c]Department of Applied Chemistry, Faculty of Engineering, Aichi Institute of Technology, 1247, Yachigusa, Yakusa-cho, Toyota, Aichi, 470-0392, Japan

[d]Department of Chemistry and Biochemistry, University of Kitakyushu, 1-1 Hibikino, Wakamatsu-ku, Kitakyushu, Fukuoka 808-0135, Japan

*Corresponding author
Mina Sakuragi, Associate professor
E-mail: d08b0101@nano.sojo-u.ac.jp
Tel: +8196-326-3593

__Table of Contents__

Results and Discussion

1. SAXS and SANS structural characterization
2. DLS measurements
3. Hydrophobic fluorescein skin penetration
4. SANS profiles of bicelles applied to SCs
5. SANS profiles of DPPC applied to SCs

1. SAXS and SANS structural characterization

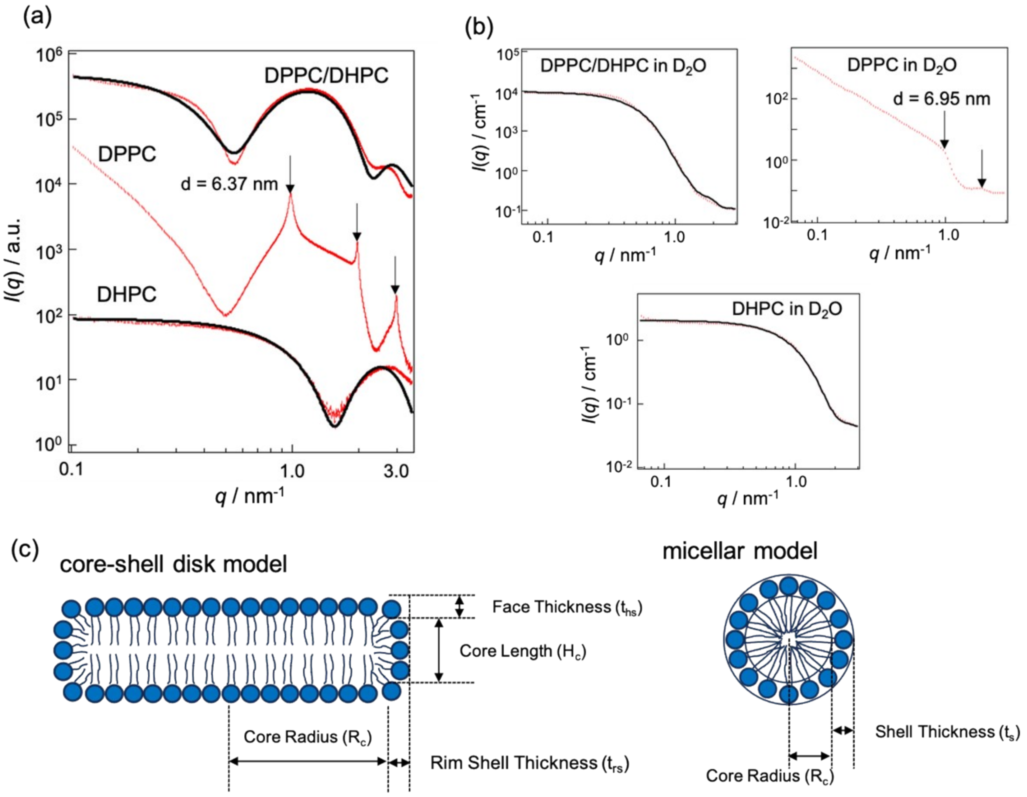

Fig. S1 a) SAXS profiles and b) SANS profiles of bicelles, DPPC dispersion, and DHPC solution. Black lines: theoretical curves. Red lines: experimental data. (c) Illustration of a core-shell disk and core shell sphere for fitting the SAXS and SANS profiles.

The experimental and theoretical curves were in good agreement. The best-fit parameters for the bicelles and DHPC micelles are shown in Table S1 and S2, respectively.

Table S1. Best fit parameters for fitting analysis of bicelle solution.

	Bicelle in H_2O (SAXS)	Bicelle in D_2O (SANS)
Core Radius (R_c) /nm	3.5	3.3
Core Length (H_c)/nm	2.8	2.8
Face Shell Thickness (t_{hs})/nm	1.4	1.2
Rim Shell Thickness (t_{rs}) /nm	0.7	0.9
SLD Core (ρ_c) / $\times 10^{-6}$Å^{-2}	8.32	-0.28
SLD Face Shell (ρ_{hs}) / $\times 10^{-6}$Å^{-2}	10.7	1.81
SLD Rim Shell (ρ_{rs}) / $\times 10^{-6}$Å^{-2}	9.9	1.75
SLD Solvent (ρ_{sol}) / $\times 10^{-6}$Å^{-2}	9.41	6.28
Radial Polydispersity	0.29	0.2

Table S2. Best fit parameters for fitting analysis of micelle solution.

	Micelle in H_2O (SAXS)	Micelle in D_2O (SANS)
Core Radius (R_c) /nm	1.3	1.34
Shell Thickness (t_s) /nm	0.5	0.5
SLD Core (ρ_c) / $\times 10^{-6}$Å^{-2}	8.32	-0.28
SLD Shell (ρ_s) / $\times 10^{-6}$Å^{-2}	11.2	1.81
SLD Solvent (ρ_{sol}) / $\times 10^{-6}$Å^{-2}	9.41	6.28
Radial Polydispersity	0.1	0.33

The best-fit parameters in SAXS and SANS for the bicelles and micelles were nearly identical between SAXS and SANS, indicating that similar structures were formed.

2. DLS measurements

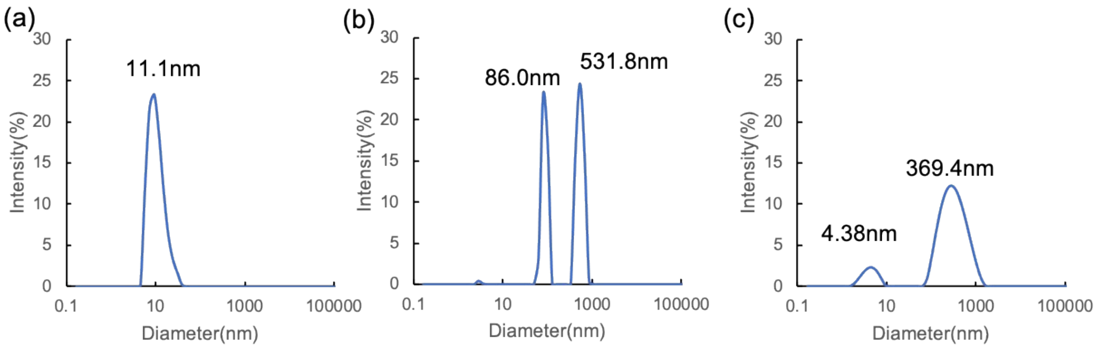

Fig. S2 Averaged diameters of (a) bicelles, (b) DPPC vesicles, and (c) DHPC micelles.

3. Hydrophobic fluorescein skin penetration

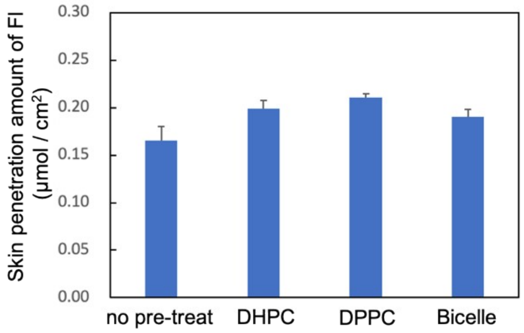

Fig. S3 Average skin penetration of hydrophobic Fl. Data are means ± SD of three experiments.

4. SANS profiles of bicelles applied to SCs

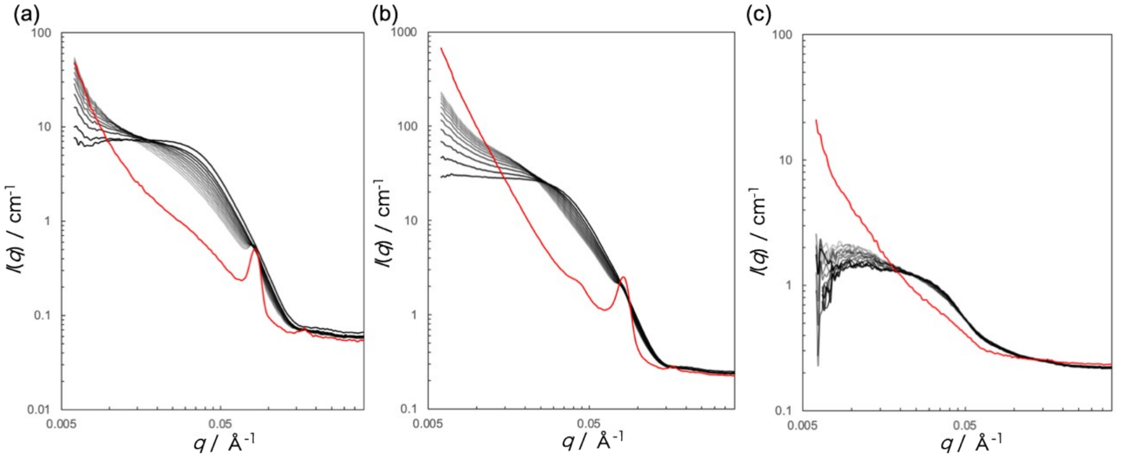

Fig. S4 SANS profiles of the SCs applying a) d-DPPC / d-DHPC bicelles, b) d-DPPC / h-DHPC bicelles, and c) h-DPPC / d-DHPC bicelles. The solvent of all samples was H_2O. The exposure time was 20 min; continuous measurements were conducted for up to 4 h, and a subsequent measurement was performed after 24 h.

5. SANS profiles of DPPC applied to SCs

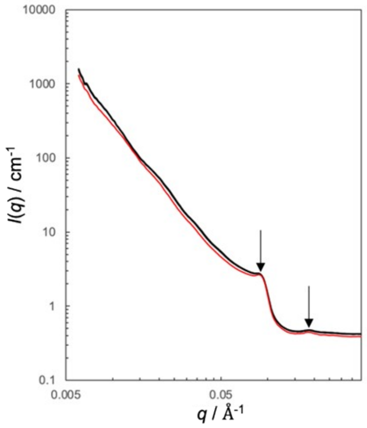

Fig. S5 SANS profiles of the SC applying d-DPPC dispersed in H_2O. The exposure time was 20 min; continuous measurements were conducted for up to 4 h, and a subsequent measurement was performed after 24 h.
